# A Novel Artificial Eagle-Inspired Optimization Algorithm for Trade Hub Location and Allocation Method

**DOI:** 10.3390/biomimetics10080481

**Published:** 2025-07-22

**Authors:** Shuhan Hu, Gang Hu, Bo Du, Abdelazim G. Hussien

**Affiliations:** 1 Department of Applied Mathematics, Xi’an University of Technology, Xi’an 710054, China; hushuhan2023@163.com; 2 Unmanned System Research Institute, Northwestern Polytechnical University, Xi’an 710072, China; dubo2022@sina.com; 3 Department of Computer and Information Science, Linköping University, 581 83 Linköping, Sweden; abdelazim.hussien@liu.se; 4 Faculty of Science, Fayoum University, Faiyum 63514, Egypt

**Keywords:** trade hub location, low-cost transportation, intelligent optimization algorithm, artificial eagles

## Abstract

Aiming for convenience and the low cost of goods transfer between towns, this paper proposes a trade hub location and allocation method based on a novel artificial eagle-inspired optimization algorithm. Firstly, the trade hub location and allocation model is established, taking the total cost consisting of construction and transportation costs as the objective function. Then, to solve the nonlinear model, a novel artificial eagle optimization algorithm (AEOA) is proposed by simulating the collective migration behaviors of artificial eagles when facing a severe living environment. Three main strategies are designed to help the algorithm effectively explore the decision space: the situational awareness and analysis stage, the free exploration stage, and the flight formation integration stage. In the first stage, artificial eagles are endowed with intelligent thinking, thus generating new positions closer to the optimum by perceiving the current situation and updating their positions. In the free exploration stage, artificial eagles update their positions by drawing on the current optimal position, ensuring more suitable habitats can be found. Meanwhile, inspired by the consciousness of teamwork, a formation flying method based on distance information is introduced in the last stage to improve stability and success rate. Test results from the CEC2022 suite indicate that the AEOA can obtain better solutions for 11 functions out of all 12 functions compared with 8 other popular algorithms. Faster convergence speed and stronger stability of the AEOA are also proved by quantitative analysis. Finally, the trade hub location and allocation method is proposed by combining the optimization model and the AEOA. By solving two typical simulated cases, this method can select suitable hubs with lower construction costs and achieve reasonable allocation between hubs and the rest of the towns to reduce transportation costs. Thus, it is used to solve the trade hub location and allocation problem of Henan province in China to help the government make sound decisions.

## 1. Introduction

As a classical problem in the field of operation research optimization, the location problem is widely studied in production and daily life, logistics, and even military contexts, such as the location of factories, warehouses, emergency centers, fire stations, garbage disposal centers, logistics centers, and the deployment of the phasor measurement units [1,2]. Especially with the acceleration of urbanization and rapid economic development, the establishment of trade hubs among towns directly affects the service mode, service quality, service efficiency, service cost, etc., thus affecting profits and market competitiveness, and even determining the fate of towns [3,4,5]. A suitable choice of locations will bring convenience to people’s lives, reduce costs, and expand profits and market share. Therefore, studying the trade hub location problem has been a research hotspot with significant economic, social, and military significance.

Compared with the traditional facility location problem, when determining the location of hubs, it is necessary to decide on the allocation relationship between hubs and non-hub nodes [6]. Generally, the allocation relationship between a hub and non-hub nodes can be divided into single and multiple allocations [7]. A single allocation means that a non-hub node can only be allocated to one hub, while the latter implies that a non-hub node can be assigned to multiple hubs [8]. Therefore, the problem of hub location is more complicated than that of the traditional facility location. To solve this key and challenging problem, scholars have carried out a lot of in-depth research. Early research on the hub location method mainly focused on a continuous model, a qualitative analysis pattern [9]. However, this method faces many drawbacks, such as a single structural system and a smaller scope. Meanwhile, with the development of computers and the advent of the digital age, the discrete hub location method based on quantitative analysis has been widely used, mainly by establishing an objective function and seeking its optimal solution to determine the location of hubs [10]. Due to the difficulty of solving large-scale models, various mathematical algorithms are adopted instead of initial enumeration methods. For example, Telmo proposed an improved initial dual algorithm based on utilizing sequential and parallel strategies to solve capacity-constrained *p*-hub allocation problems. Its advantage was proved by the results carried out on a standard testbed [11]. Rostami et al. studied the single assignment hub location problem under demand uncertainty and applied an alternating convex mixed-integer nonlinear programming method for the established model [12]. When the dimension of models is further increased, some optimization methods are employed to overcome the insufficient accuracy. Zhang et al. studied the aviation hub location optimization problem of an express enterprise, primarily using a *p*-median model and a greedy dropping heuristic algorithm to solve the initial candidate locations of the aviation hubs [13]. Silva and Cunha proposed a tabu search heuristic algorithm to solve the uncapacitated single allocation *p*-hub maximal covering problem [14]. For the deployment problem of the phasor measurement units (PMUs), Theodorakatos et al. proposed a multi-objective optimization function minimized under a set of 0–1 Boolean observability constraints and used a designed binary particle swarm optimization (BPSO) to effectively solve the model [15]. Singh presented a binary gravitational search algorithm (BGSA) methodology for the optimal placement of phasor measurement units to minimize the total number of PMUs installed at various buses [16]. Rathore et al. verified the efficacy of diversification-based learning (DBL) in expediting the performance of simulated annealing (SA) in hub location problems. Results showed that the proposed method outperforms the original SA algorithm in terms of accuracy and convergence rates [17]. To obtain lower service costs, Bhattacharjee and Mukhopadhyay further proposed a refined version of the genetic algorithm to find a suitable network of demand nodes. Better solutions over other meta heuristics were gained for more than 88% of cases [18].

In the existing methods, intelligent optimization algorithms (IOAs) have been regarded as an effective approach to solving hub location problems. This is because this kind of algorithm has an advantage of a simple structure, wide applicability, and reliable search performance compared with the traditional methods [19]. IOAs belong to random search methods, which continuously optimize the solution based on specific search mechanisms by simulating biological evolution, group behavior, and mathematical theory [20]. Current intelligent optimization algorithms are generally divided into three categories: evolution-based algorithms, swarm-based algorithms, and mathematical-physical theory-based algorithms [21]. The genetic algorithm (GA) and the differential evolution (DE) algorithm are the representatives of evolution-based algorithms [22,23]. They search for the best solution in decision spaces by modeling the mechanism of heredity, variation, and elimination in population evolution. The swarm-based algorithms are inspired by cooperative behaviors when hunting, defending, and migrating in the population, such as the classical particle swarm optimization (PSO) algorithm [24] and the whale optimization algorithm (WOA) [25]. The last class discovers effective searching strategies by examining classical mathematical or physical theorems, such as the gradient-based optimizer (GBO) [26] and the multi-verse optimizer (MVO) [27].

However, although various IOAs have emerged in recent years, there are still some deficiencies when facing complex engineering optimization problems. When there are many local optimums of the objective function, IOAs are easily misled and miss the global optimal solution, further leading to lower convergence speed and solving accuracy. When facing optimization problems with various characters, the ability of IOAs needs to be improved to form general applicability. Therefore, scholars proposed improved IOAs to overcome these disadvantages, such as the opposition learning strategy [28], the wavelet mutation mode [29], the Levy flight method [30], collaborative search thinking [31], etc.

However, due to the structural design of the algorithm itself, the introduction of improvement strategies has a limited effect on the performance of IOAs. Thus, this paper proposes a novel artificial eagle optimization algorithm (AEOA) by simulating the collective migration behavior of artificial eagles when faced with severe living environments. This novel algorithm is also employed to solve the established trade hub location and allocation model to provide a reference scheme for decision makers. The main contributions of this paper are as follows.

(1) A trade hub location and allocation model among towns with various economic levels is established. This model has an optimization target of the total cost, consisting of the hub’s construction cost and the transportation cost between cities and towns.

(2) A novel intelligent optimization algorithm (AEOA) is proposed. The artificial eagle population has three key stages that help the algorithm obtain solutions with higher accuracy and avoid local optimums.

(3) According to the results of the CEC2022 test suite, quantitative analysis, convergence analysis, and stability analysis are performed to measure the performance of the proposed AEOA.

(4) The trade hub location and allocation method based on the AEOA is introduced. Results from two simulated cases and a real-world case for Henan province in China prove that the method is reasonable and reliable, reducing the total cost by selecting optimal hub locations.

Section 2 describes the trade hub location and allocation problem in the remainder of the paper and establishes the corresponding optimization model. Section 3 proposes a novel intelligent algorithm (AEOA) and illustrates the detailed implementation process. Test results and analysis of the AEOA are provided in Section 4 to measure its performance. In Section 5, the AEOA is employed to solve the trade hub location and allocation model. It verifies its ability by being tested on two simulated cases and is further applied to solve the choice of hubs among 36 towns with various gross domestic products in Henan province, China. Finally, the conclusion and future work are presented in Section 6.

## 2. Establishment of the Trade Hub Location and Allocation Model

### 2.1. Problem Description

**Figure 1:**
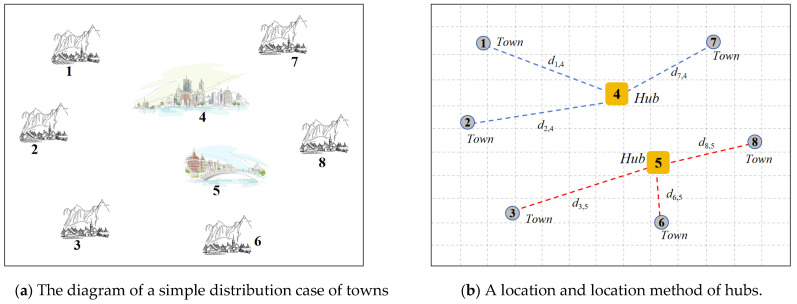
The schematic diagram of the trade hub location problem.

This paper focuses on the location and allocation of trade hubs among cities and towns with different levels of development. For example, Figure 1a shows a diagram of this kind of problem. On the map, eight towns are randomly distributed, among which numbers 4 and 5 are large cities with prosperous economies, while the rest are small towns. The government plans to build two large trade hubs in this area to facilitate transport between towns and revitalize the local economy. Therefore, each town might be selected to play a different role, either as a trading hub or as an ordinary town affiliated with a trade hub. The choice of trade hubs needs to consider construction costs and transportation costs between hubs and their affiliated towns. For construction costs, a better economic situation will lead to less expenditure on materials, resulting in lower construction costs. Meanwhile, trade hubs should be located centrally among towns to reduce transportation costs. Thus, Figure 1b presents the selection results of hubs under ideal conditions. Two big cities, 4 and 5, are selected as the two trade hubs to optimize these ideal conditions.

However, these ideal and reasonable conditions are not common. When the economic level of each town is similar, or a developed town is at the edge, this problem becomes intractable.

**Figure 2:**
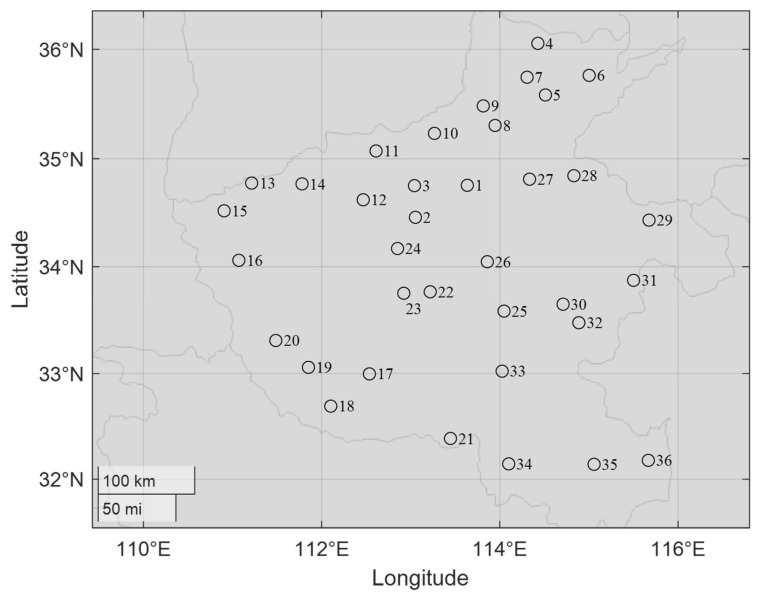
The map of the main towns of Henan province in China.

For example, Figure 2 maps the main towns in Henan province in China. Table 1 lists the gross domestic product (GDP) of those main towns of Henan province in 2022, representing their level of economic development to measure the cost of building a hub in the local area. Thus, choosing reasonable locations among these important towns in Henan province to establish trade hubs, facilitate transportation, and reduce costs is the main focus of this paper.

### 2.2. The Mathematical Model of the Trade Hub Location and Allocation Problem

To select the most suitable towns as trade hubs, this problem is regarded as an optimization model with constraint conditions to solve. The total number of towns is set to *M*, and *P* hubs must be selected and constructed (*P* < *M*). The objective of the optimization model is to reduce construction and transportation costs while ensuring that the affiliated towns are associated with the selected *P* hubs. Thus, the optimization model can be established as follows.

(1) $$\begin{array}{l} \min\;f(X)=w_{1}\cdot \sum_{i=1}^{M}\sum_{j=1,j\neq i}^{M}\left(d_{i,H(i)}c_{i,H(i)}+\alpha d_{H(i),H(j)}c_{H(i),H(j)}+d_{H(j),j}c_{H(j),j}\right)+w_{2}\cdot \sum_{i=1}^{M}x_{i,i}\cdot F_{i}\\ s.t.\;\left\{\begin{array}{l} h(X)=\sum_{i=1}^{M}x_{i,i}=P,\\ g(X)=\sum_{j=1}^{M}x_{i,j}=1,\;j=1,\;2,\;\ldots ,\;M,\\ H=[h_{1},h_{2},\ldots ,h_{M}],\;h_{i}\in \{1,\;2,\;\ldots ,\;M\},\\ x_{i,j}\in \{0,\;1\}. \end{array}\right. \end{array}$$

where $X=\left[\begin{array}{cccc} x_{1,1} & x_{1,2} & \ldots & x_{1,M}\\ x_{2,1} & x_{2,2} & \ldots & x_{2,M}\\ \vdots & \vdots & \ddots & \vdots \\ x_{M,1} & x_{M,2} & \ldots & x_{M,M} \end{array}\right]$ is a *M* × *M* matrix with decision variables 0 or 1, determining the trade hubs and their affiliated towns. If *x_i_*_,*i*_ = 1, the *i* th town is set as a trade hub. If *x_i_*_,*j*_ = 1 (*i* ≠ *j*), it means town *j* is affiliated with the *i* th town (trade hub). *d_i_*_,*j*_ and *c_i_*_,*j*_ are the distance and freight rate between the *i* town and *j* town. *F_i_* is the construction cost of the *i* town related to the level of economic development. *α* = 0.7 is a discount factor for the freight between hubs. ***H*** is an integer vector, indicating to which trading hub each town is affiliated. *h*(***X***) and *g*(***X***) are two constraints; the former ensures that the number of trade hubs is *P*, while the latter ensures that each town can only belong to one hub. *w*_1_ = 5 and *w*_2_ = 1 are two fixed parameters that balance construction and transportation costs.

For example, in the case shown in Figure 1b, the final decision variable is as follows: $X=\left[\begin{array}{cccccccc} 0 & 0 & 0 & 0 & 0 & 0 & 0 & 0\\ 0 & 0 & 0 & 0 & 0 & 0 & 0 & 0\\ 0 & 0 & 0 & 0 & 0 & 0 & 0 & 0\\ 1 & 1 & 0 & 1 & 0 & 0 & 1 & 0\\ 0 & 0 & 1 & 0 & 1 & 1 & 0 & 1\\ 0 & 0 & 0 & 0 & 0 & 0 & 0 & 0\\ 0 & 0 & 0 & 0 & 0 & 0 & 0 & 0\\ 0 & 0 & 0 & 0 & 0 & 0 & 0 & 0 \end{array}\right]$. That is, towns 4 and 5 are set as trade hubs. The relation between trade hubs and affiliated towns can be described by the integer vector $H=[h_{1},h_{2},\ldots ,h_{M}]=[4,\;4,\;5,\;4,\;5,\;5,\;4,\;5]$. Trade hub 4 will conduct direct trade with affiliated towns 1, 2, and 7. When town 1 needs to conduct trade with town 7, the transportation cost is calculated by $d_{1,4}c_{1,4}+\alpha d_{4,4}c_{4,4}+d_{4,7}c_{4,7}$. When town 1 needs to conduct trade with town 3, it will utilize hubs 4 and 5. Thus, the transportation cost is calculated by $d_{1,4}c_{1,4}+\alpha d_{4,5}c_{4,5}+d_{5,3}c_{5,3}$.

However, when the number of towns increases substantially and their economic levels are close, the model shown in Equation (1) is complex and difficult to solve. Thus, intelligent optimization algorithms are considered for solving this problem reasonably and efficiently.

## 3. The Artificial Eagle Optimization Algorithm

This section proposes a novel intelligent algorithm named artificial eagle optimization to solve various engineering optimization problems. The inspiration and the specific implementation process are provided to clearly illustrate the characteristics of the proposed algorithm.

### 3.1. Motivation for the Construction of the Artificial Eagle Optimization Algorithm

Eagles are broadly defined as small- to medium-sized diurnal raptors characterized by curved beaks, powerful talons, strong wings, and robust skeletons. Despite their diverse species and extensive habitats, eagles face significant survival challenges as human settlements continue to expand. Consequently, humans have intervened in the eagle’s breeding process to avoid the risk of extinction [32]. That is, artificial eagles in this paper refer to the eagles that have been artificially incubated, bred, and trained. Artificial eagles have the following characteristics compared with wild eagles:

(1) Intelligent thinking ability. Through artificial training, eagles can understand the pattern of human thinking through extensive positive or negative feedback. Especially when facing increasingly severe living environments, this kind of intelligent thinking method can help eagles accurately analyze the current situation and make correct judgments to find a more suitable habitat.

(2) Teamwork ability. Usually, wild eagles are solitary animals. However, artificial eagles are more likely to act in groups, such as migration, hunting, or communication. In this way, eagles can assist one another to enhance the viability of the whole population. This paper mainly focuses on the ability of artificial eagles to maintain formation during migration, which can strengthen cooperation and communication between populations and improve the chances of reaching suitable habitats.

(3) Keen observation skills. Though artificial eagles are bred and grown under artificial intervention, they still retain their unique keen observation skills. Whether in the stage of feeding or migration, this ability helps eagles fully perceive the environment and make predictions in advance to fly in the right direction.

Thus, after considering the above characteristics, this paper constructs a new algorithm to search for the optimal solution by simulating the collective migration behavior of artificial eagles when they are faced with a severe living environment. The specific implementation steps of the AEOA are shown below.

### 3.2. The Population Initialization Stage

Equation (2) shows the typical formulation of a single objective minimization problem with *dim* decision variables.

(2) $$\begin{array}{l} \min\;f(X^{*})=f([x_{1}^{*},\;x_{2}^{*},\;\ldots ,\;x_{dim}^{*}])\\ s.t.\;x_{i}^{\min}\;\leq \;x_{i}^{*}\;\leq \;x_{i}^{\max}\;, \end{array}$$

where $X^{*}=[x_{1}^{*},\;x_{2}^{*},\;\ldots ,\;x_{dim}^{*}]$ represents a candidate solution for the optimization problem, and $f(X^{*})$ is the fitness value corresponding to the current candidate solution. A smaller fitness value indicates a solution with higher accuracy. $Lb=[x_{1}^{\min},\;x_{2}^{\min},\;\ldots ,\;x_{dim}^{\min}]$ and $Ub=[x_{1}^{\max},\;x_{2}^{\max},\;\ldots ,\;x_{dim}^{\max}]$ are the lower and upper bounds that variables need to satisfy.

The first step of the AEOA is to generate *N* candidate solutions randomly to form an initial population ***Pop*** of artificial eagles, which has the following formulation.

(3) $$Pop=\left[\begin{array}{c} Pos_{1}\\ Pos_{2}\\ \vdots \\ Pos_{N} \end{array}\right]=\left[\begin{array}{cccc} x_{1,1} & x_{1,2} & \ldots & x_{1,dim}\\ x_{2,1} & x_{2,2} & \ldots & x_{2,dim}\\ \vdots & \vdots & \ddots & \vdots \\ x_{N,1} & x_{N,2} & \ldots & x_{N,dim} \end{array}\right],$$

where $Pos_{i}$ represents the position of the *i* th artificial eagle corresponding to the candidate solution $X^{*}$ shown in Equation (2). Then, each ***Pos****_i_* is calculated by Equation (4).

(4) $$Pos_{i}=Lb+rand\cdot (Ub-Lb),1\;\leq \;i\;\leq \;N,$$

where *rand* is a random number between 0 and 1.

### 3.3. Situational Awareness and Analysis Stage

By analyzing the characteristics of artificial eagles, their intelligent thinking ability will be further enhanced under manual interventions. In the migration process, this feature is reflected in the awareness and thinking of the current situation of the whole population. Each artificial eagle will update its position by analyzing information about other eagles in better positions. Firstly, the positions of artificial eagles can be ranked based on their fitness values. For the *i*-th artificial eagle, its position ***Pos****_i_* will be affected by other three optimal positions within the current population, denoted as ***Pos****_α_*, ***Pos****_β_*, and ***Pos****_γ_* (*α*, *β*, *γ ≠ i*). Then, a potentially more suitable location will be generated by Equation (5).

(5) $$NewPos_{i}(t)=Pos_{\alpha }(t)\pm S\cdot (Pos_{\beta }(t)\pm Pos_{\gamma }(t)),$$

where *t* and *T* are the current iteration and the maximum iteration, respectively. S=1.2−t/T is an attenuation coefficient that decreases with the number of iterations. In the early stage of searching (the iterative number *t* is relatively small), a larger *S* helps the algorithm explore the feasible space more quickly and get closer to the theoretically optimal solution. A smaller *S* can maintain the overall population quality in the later stage of searching (the iterative number *t* is relatively bigger). The positive and negative signs in Equation (5) are randomly determined to reflect the randomness of the thinking process.

Then, the greedy selection strategy is used to help the artificial eagle choose better positions after thinking and analysis, as shown in Equation (6).

(6) $$\left\{\begin{array}{l} Pos_{i}(t+1)=Pos_{i}(t),\;\mathrm{if}\;f(Pos_{i}(t))<f(NewPos_{i}(t)),\\ Pos_{i}(t+1)=NewPos_{i}(t),\;\mathrm{if}\;f(Pos_{i}(t))\geq f{\left({NewPos}_{i}(t)\right)}_{i}, \end{array}\right.$$

where *f* (***Pos****_i_*(*t*)) and *f* (***NewPos****_i_*(*t*)) are the fitness values of ***Pos****_i_*(*t*) and ***NewPos****_i_*(*t*), respectively.

### 3.4. Free Exploration Stage

During the migration, each artificial eagle also has a particular ability to update its position autonomously. For example, it may move to a new candidate location, relying solely on the best position information and random step length or direction obtained by the current environment feedback. This behavior reflects the fast exploration ability of the optimal solution in the complex decision space. Thus, two modes are set to simulate this stage. Equation (7) shows the first mode, where artificial eagles fly around the optimal position based on the Levy flight pattern.

(7) $$Pos_{i}(t+1)=Pos_{i}(t)+levy\cdot (Pos_{best}(t)-Pos_{i}(t)),$$

where *levy* is a random value following the Levy distribution. ***Pos****_best_*(*t*) is the position of the artificial eagle with the lowest fitness value, noted as the optimal position closest to the migration destination.

However, this search method relies too much on the current optimal position. If the current best position only considers the local situation rather than the global one, the corresponding local optimal solution in the optimization problem will significantly affect the solution accuracy. Therefore, another method with stronger randomness is applied, as shown in Equation (8).

(8) $$Pos_{i}(t+1)=(Pos_{p}(t)-rand\cdot Pos_{ave}(t)),$$

where $Pos_{ave}(t)=\left(Pos_{r1}(t)+Pos_{r2}(t)+Pos_{r3}(t)\right)/3$ is the average of three randomly selected positions ***Pos****_r_*_1_(*t*), ***Pos****_r_*_2_(*t*), and ***Pos****_r_*_3_(*t*). ***Pos****_p_*(*t*) is obtained by combining the average position and the optimal position, which is achieved as follows:

(9) $$Pos_{p}(t)=\beta \cdot Pos_{best}(t)+(1-\beta )\cdot Pos_{ave}(t).$$

where *β* is a random number between 0 and 1. The above two modes will be executed with equal probability in the algorithm, as shown in Equation (10).

(10) $$\left\{\begin{array}{l} Pos_{i}(t+1)=Pos_{best}(t)\cdot levy+Pos_{i}(t),\;\mathrm{if}\;rand>0.5,\\ Pos_{i}(t+1)=(Pos_{p}(t)-rand\cdot Pos_{ave}(t)),\;\mathrm{if}\;rand\leq 0.5. \end{array}\right.$$

### 3.5. Flight Formation Integration Stage

**Figure 3:**
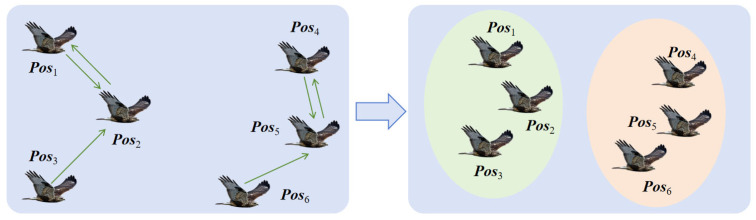
Position update pattern based on the nearest distance.

**Figure 4:**
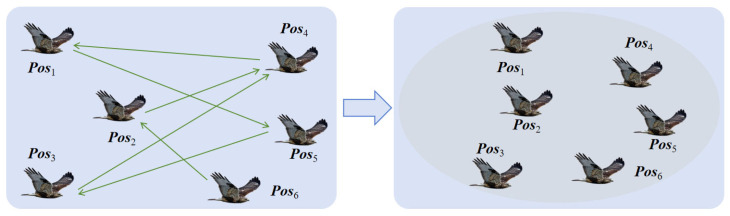
Position update pattern based on the far distance.

Due to manual training, artificial eagles also have a consciousness of teamwork while exploring freely. Thus, a novel formation flying method based on distance information is adopted to reflect the flight formation integration behavior. Firstly, a distance vector $Dis_{i}(t)=[Dis_{i,1},Dis_{i,2},\ldots ,Dis_{i,N}]$ needs to be calculated to record the distance between individual position ***Pos****_i_* and others. Then, by sorting the distance from smallest to biggest, an ordered distance vector can be further determined. The *i* th artificial eagle will update its position based on the ordered distance vector by selecting the more distant position as the target, noted as ***Target****_i_*. Figure 3 and Figure 4 illustrate the intention and effect of this position update pattern. In Figure 3, artificial eagles select the nearest positions as targets. In this way, artificial eagles tend to form several small groups together rather than migrating en masse to more suitable areas. In Figure 4, each artificial eagle chooses to move farther away, effectively ensuring that the entire population moves synergistically to more appropriate places, ensuring searching stability.

After determining the target, the *i* th artificial eagle will update the position as shown in Equation (11).

(11) $$\left\{\begin{array}{l} Pos_{i}(t+1)=Pos_{i}(t)+R\cdot (Target_{i}-I\cdot Pos_{i}(t)),\;\mathrm{if}\;f(Target_{i})<f(Pos_{i}(t)),\\ Pos_{i}(t+1)=Pos_{i}(t)+R\cdot (Pos_{i}(t)-Target_{i}),\;\mathrm{if}\;f(Target_{i})\geq f(Pos_{i}(t)). \end{array}\right.$$

where ***R*** is a vector with random elements from 0 to 1.

**Figure 5:**
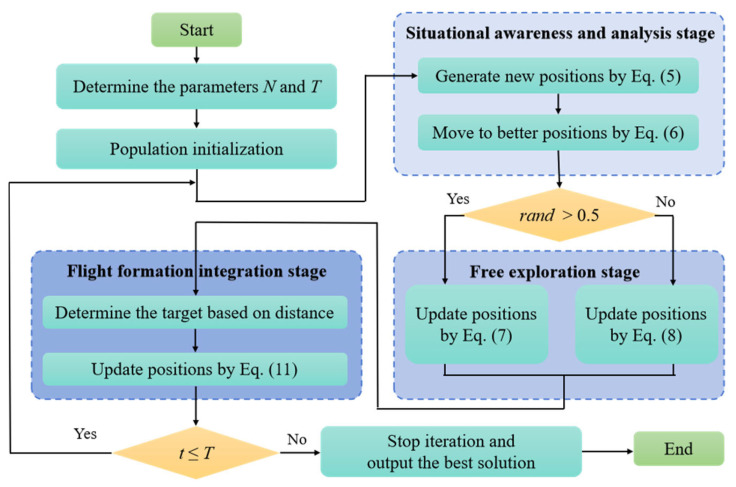
The flow chart of the AEOA.

Based on the above stages, Figure 5 draws the whole flow chart of the AEOA for reference and implementation.

## 4. Experimental Results and Analysis of the Proposed AEOA

This section verifies the comprehensive ability of the proposed AEOA by solving a series of typical test functions. Some popular intelligent optimization algorithms are also selected as comparative experiments to illustrate whether the AEOA is competitive.

### 4.1. Experimental Design and Comparison Algorithms

The CEC2022 test suite is a popular test tool applied to verify the performance of various optimization algorithms [33]. It comprises 12 functions, which are divided into four types, namely uni-modal functions (F01), multi-modal functions (F02–F05), hybrid functions (F06–F08), and composition functions (F09–F12). Table 2 provides detailed information on the CEC2022 test suite. Different types of functions can effectively test the accuracy, convergence, and stability of intelligent optimization algorithms.

Then, other eight classical and popular intelligent optimization algorithms are employed as comparison algorithms, which are the golden Jackal optimization (GJO) algorithm [34], the Harris Hawks optimization (HHO) algorithm [35], the beluga whale optimization (BWO) algorithm [36], the walrus optimizer (WO) [37], the Chinese pangolin optimizer (CPO) [38], the particle swarm optimization (PSO) algorithm [39], the Newton–Raphson based optimizer (NRBO) [40], and the sand cat swarm optimization (SCSO) algorithm [41]. Among these algorithms, the PSO and the HHO algorithm are representatives of classical methods, which have been successfully applied to various engineering optimization problems, while the others have been proposed in the last three years and have been reviewed and published in well-known journals. In addition, the types of algorithms are also considered. The NRBO is constructed by analyzing the mathematical theorems, while the others are inspired by hunting or cooperative behaviors among social animals. Thus, compared with the above algorithms, the proposed AEOA will be tested fairly and objectively. Table 3 shows the key parameters of the comparison algorithm.

Finally, the AEOA and other eight comparison algorithms are used to solve test functions of CEC2022. All algorithms have the same initial parameters, which are population size *N* = 100, maximum iterations *T* = 1000, and the times of independent executions *Run* = 30. After running 30 times, each algorithm will obtain 30 solving solutions for each test function. Based on the obtained data, the following evaluation indicators are used to measure algorithm’s performance.

(1) Average fitness value (Ave_f). This evaluation indicator is obtained by calculating the average value of 30 solving solutions. It can measure algorithm’s accuracy objectively and reasonably, avoiding the influence of other accidental factors.

(2) The standard deviation (Std_f). This evaluation indicator is the standard deviation of the results after 30 runs. It can reflect the degree of dispersion among a series of data, which is an effective tool to describe the stability of algorithms.

(3) The ranking result (Rank). The above two indicators determine this. Algorithms with smaller average fitness values have smaller ranking results. If two algorithms have the same average fitness values, the smaller standard deviation means a smaller ranking result.

(4) The average rank (Average rank) and the final rank (Final rank). The average value of ranking results on 12 test functions is employed to evaluate algorithm’s comprehensive performance. The average rank obtains the final ranking result.

### 4.2. Quantitative Analysis

Table 4 contains the solving results of the AEOA and other comparison algorithms for the CEC2022 test suite. The obtained best data on each test function is identified in bold to highlight. Among 12 test functions, the AEOA obtains better solutions with higher accuracy for 11 test functions. That is, due to its unique search mechanism, the proposed AEOA can get closer to the theoretically optimal solutions for various optimization problems. Observing the final rank, the AEOA ranks first, while the WO ranks second. However, the WO is dominant for only one test function, which helps verify the AEOA’s performance. Table 5 provides the results of the Wilcoxon rank sum test between the AEOA and comparison algorithms to verify whether the AEOA has obvious advantages over other algorithms from a statistical view. The signs ‘+’ and ‘−’ indicate that the AEOA is significantly better and worse than other algorithms, respectively. The sign ‘=’ means the *p* values between the AEOA and others are less than 0.05, indicating that there are no significant differences between algorithms. Firstly, comparing the AEOA and the WO, the AEOA performs significantly better than the WO in nine functions, while showing weaker performance only on F10. For the GJO and HHO algorithms, the AEOA has significant advantages on 11 functions. In addition, the AEOA’s performance is better than that of other algorithms, being obviously superior for all test functions. That is, the integration of multiple search stages in the AEOA is effective, enabling it to balance the global and local searching capabilities and obtain solutions with higher accuracy.

### 4.3. Convergence Analysis

**Figure A1:**
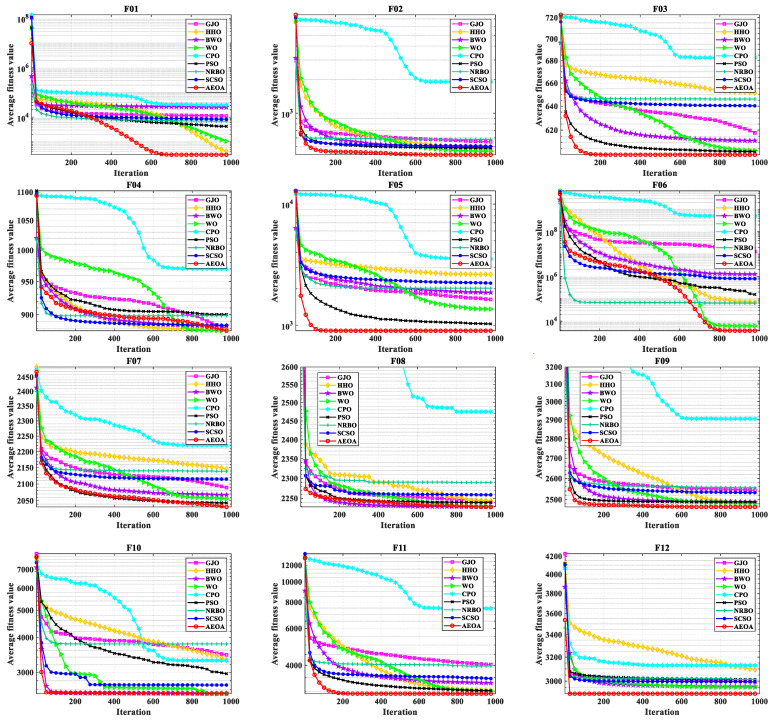
The iteration curves of the AEOA and comparison algorithms.

Figure A1 shows the iteration curves of the AEOA and other comparison algorithms, illustrating the convergence performance for test functions with various characters. In Figure A1, the curves are plotted by calculating the average values of 30 results per iteration. When the curve descends quickly, it means the corresponding algorithm has a faster convergence speed and is not affected by local optimal solutions. Except for F04 and F06, the AEOA can quickly advance in significantly fewer operations. This is because the AEOA maintains the overall migration formation while adopting the free search strategy, thus making the whole population close to the theoretical optimal solution and speeding up the convergence speed. For F04 and F06, though the SEOA is at a disadvantage in the early stage of searching, its iteration curve continues to decline later to obtain a solution with higher precision. This advantage is attributed to the flight formation integration stage, which can enhance candidate solutions’ continuous integration and update. It helps the algorithm to find solutions closer to the optimum even in the later stages of searching rather than becoming trapped in local optima.

### 4.4. Stability Analysis

**Figure A2:**
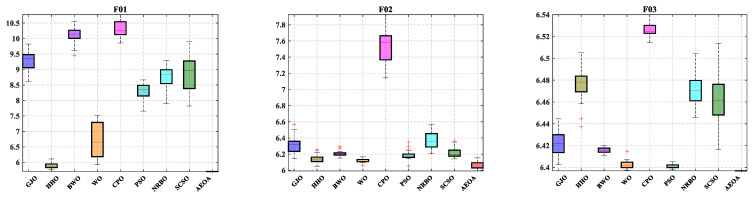

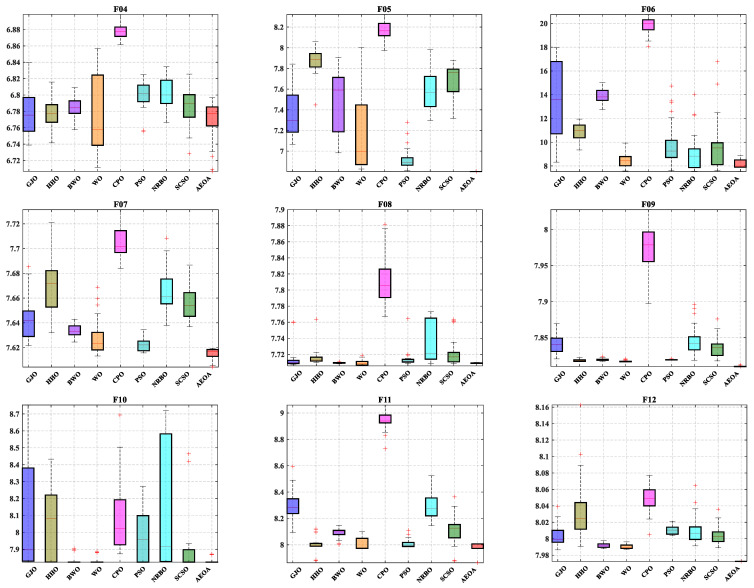
The boxplots of the AEOA and comparison algorithms.

In addition to the solution’s accuracy, the stability of algorithms is also worthy of attention in engineering optimization problems. Figure A2 draws the boxplots of all test algorithms based on the data obtained after solving the CEC2022 test suite 30 times. Combining the results in Table 3, though the AEOA is slightly inferior to the WO algorithm in solving accuracy, its stability is stronger. That is, the distribution of solving results of AEOA is concentrated, meaning it is less affected by other accidental factors. Meanwhile, the stability of WO algorithm is poor on F01, F04, and F05, though its overall performance ranks second. Thus, when facing optimization problems with complex decision spaces, the excellent stability of AEOA will help decision makers achieve better solutions at a low cost of execution times.

## 5. Trade Hub Location and Allocation Method Based on the AEOA

According to the results and analysis in Section 4, the AEOA can be used as a reliable optimization method with faster convergence speed, higher accuracy, and stronger stability. Thus, this section will introduce the AEOA into the trade hub location and allocation problem to deal with this kind of intractable task.

### 5.1. The Combination Between the Trade Hub Location and Allocation Model and the SEOA

In Section 2.2, the trade hub location and allocation problem has been established as a typical optimization model. The key to the combination of optimization algorithm and model lies in the introduction of decision variables and the calculation of the fitness value.

(1) Population initialization

In the AEOA, the population of artificial eagles represents candidate solutions to an optimization problem. Unlike simple test functions, a decision variable for the trade hub location and allocation problem among *M* towns is a matrix of *M* × *M*, where the elements are 0 or 1. Thus, the position of the *i* th artificial eagle can be expressed as follows:

(12) $$X_{i}=\left[\begin{array}{cccc} x_{1,1} & x_{1,2} & \ldots & x_{1,M}\\ x_{2,1} & x_{2,2} & \ldots & x_{2,M}\\ \vdots & \vdots & \ddots & \vdots \\ x_{M,1} & x_{M,2} & \ldots & x_{M,M} \end{array}\right],$$

where *x*_*i*,*j*_ is 0 or 1.

(2) Fitness value

Once a solution is determined, the choice of trade hubs and the connections between the remaining towns and hubs can be obtained. Then, the construction cost and total transportation cost can be calculated to form the final fitness value, as given by Equation (1).

### 5.2. Numerical Examples and Analysis

This section will test the trade hub location and allocation method based on the AEOA on two typical simulated cases and finally solve the trade hub location and allocation problem for main towns of Henan province. Meanwhile, three classical optimization algorithms are also selected as comparisons, which are the differential evolution (DE) algorithm [42], the northern goshawk optimization (NGO) algorithm [43], and the slime mold algorithm (SMA) [44].

(1) Simulated case 1

**Figure 6:**
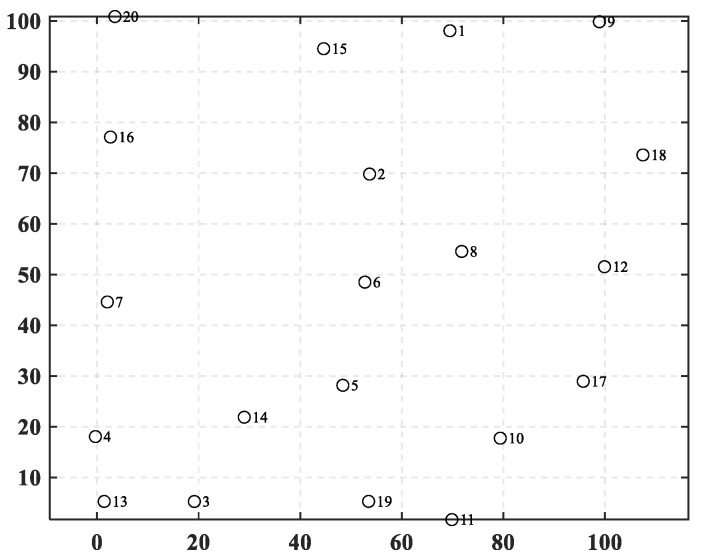
The positions of towns in case 1.

**Figure 7:**
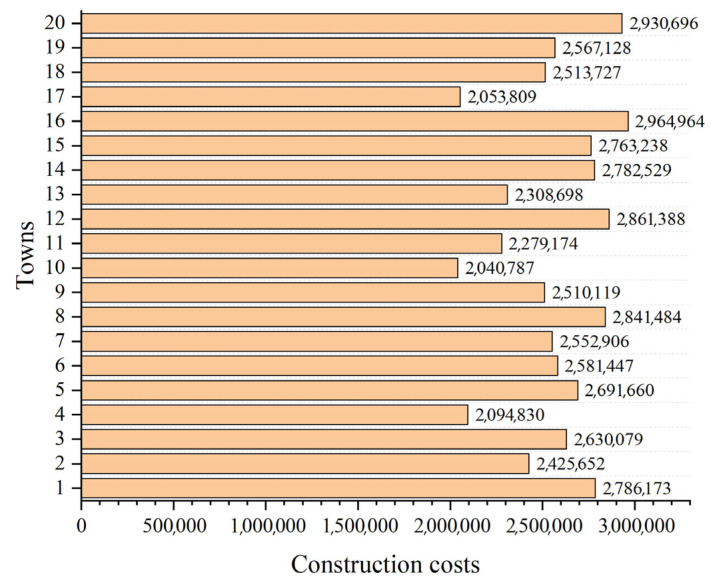
The construction costs of towns in case 1.

In this case, 20 towns are randomly distributed in a specified area, as shown in Figure 6. The information on their positions is listed in Table 6, where *x* and *y* represent the horizontal and vertical coordinates, respectively. In Figure 7, the bar chart is drawn, representing the construction costs for each town. Table A1 shows the freight cost between various towns. In this case, the freight cost is designed as an asymmetric matrix, which is intended to simulate the uncertainties in the transportation process.

**Figure 8:**
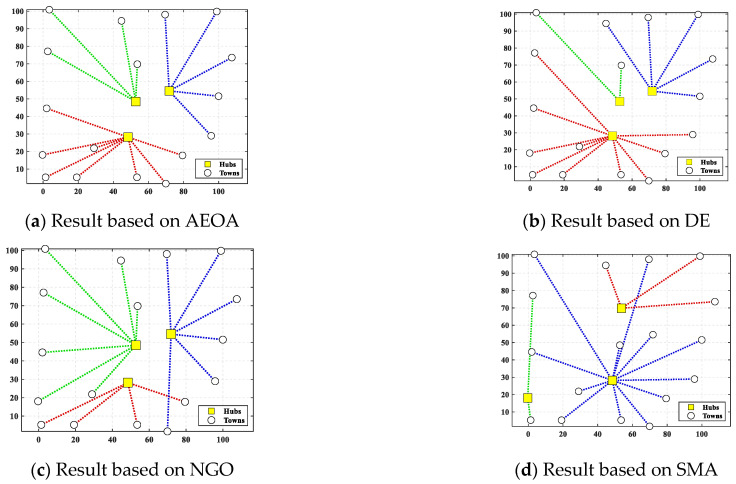
The diagram of best solutions based on different algorithms for case 1.

**Figure 9:**
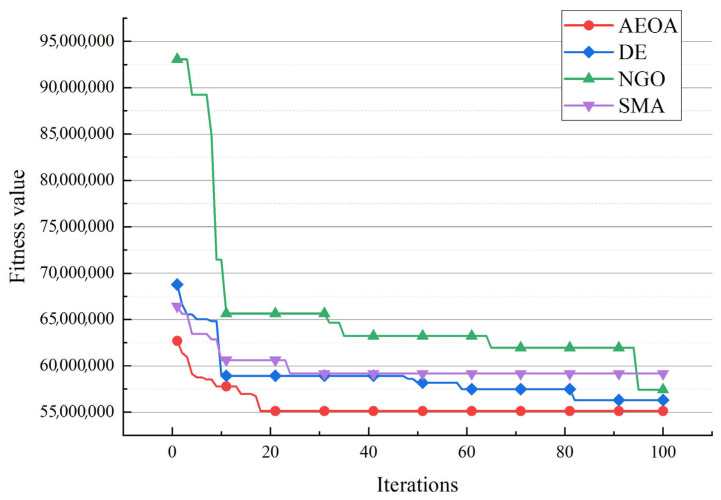
The iteration curves of different algorithms for case 1.

Table 7 shows the best solutions based on the AEOA and other algorithms. The data illustrate the choice of trade hubs and allocations of the remaining towns. For the three trade hubs in this case, they are marked as ‘—’, whereas the numbers below other towns represent which trading hubs they belong to. Figure 8 shows the corresponding diagram of the best solutions based on different algorithms. Dot lines of identical colors connect towns to their corresponding trade hub. Table 8 provides the fitness values of the best solutions to measure which algorithm performs best for solving the trade hub location and allocation problem. The AEOA, the DE algorithm, and the NGO algorithm select towns 5, 6, and 8 as the trade hubs, having the same construction costs. But for transportation cost, the allocation result based on the AEOA is more reasonable in that it fully considers the distance between hubs and towns. Thus, the final fitness value, including transportation costs and construction costs based on the AEOA, is the smallest. It proves that the proposed AEOA has excellent searching ability, which allows it to obtain better solutions through free search and formation search strategies in a complex decision space. For the SMA, it selects towns 2, 4, and 5 as trade hubs, trying to reduce the construction cost. However, the location of these hubs is not conducive to the connection with the remaining towns, resulting in a significant increase in transportation costs. Figure 9 shows the iteration curves of different algorithms for solving case 1. Obviously, the curve of the AEOA always has an advantage throughout the entire search process. That is, the AEOA can obtain better solutions after fewer iterations while other algorithms may be trapped in locally optimal solutions.

(2) Simulated case 2

**Figure 10:**
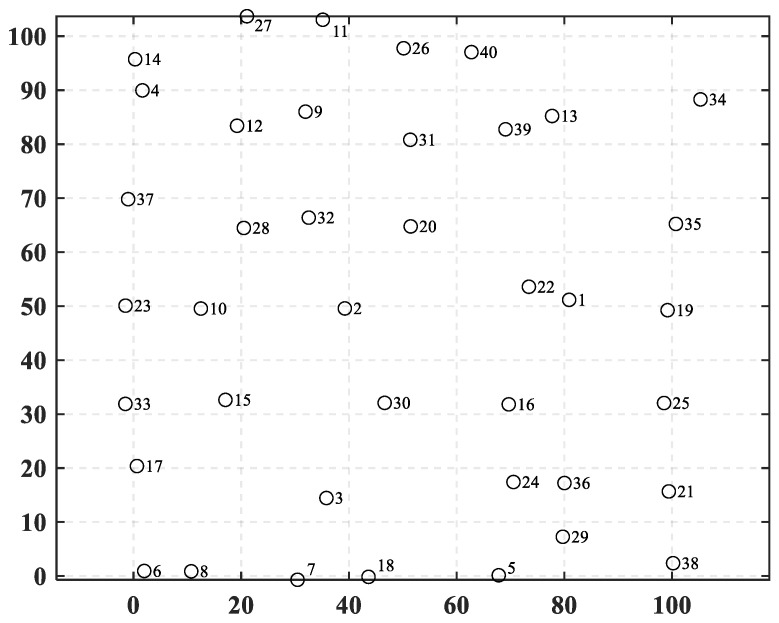
The positions of towns in case 2.

**Figure 11:**
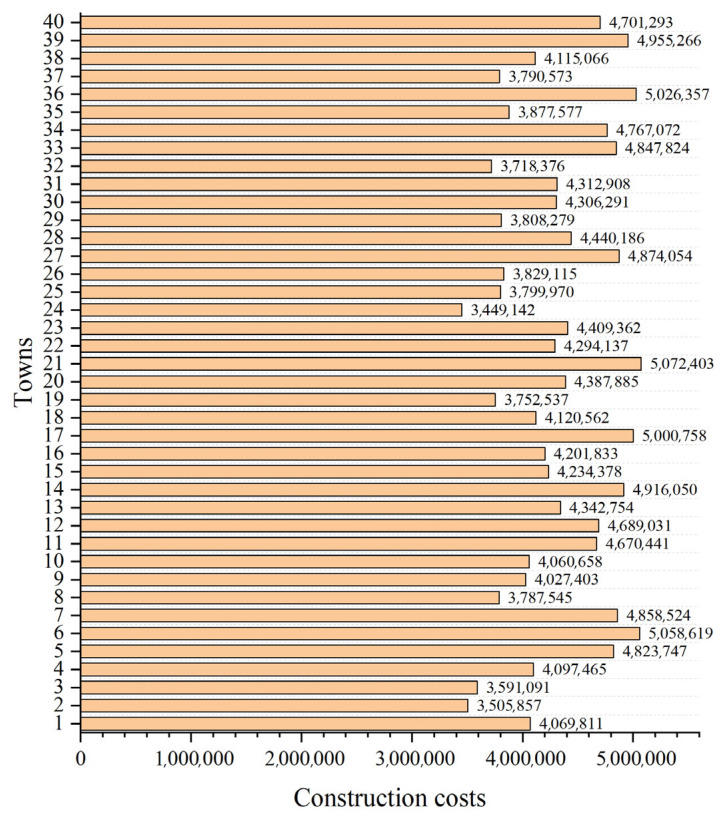
The construction costs of towns in case 2.

In this case, the number of towns is increased to 40, meaning that the complexity of a decision space is further increased. Their coordinates are shown in Table 9 and plotted in Figure 10. Figure 11 shows the randomly generated construction costs for each town. Table A2 shows the freight between each town in case 2.

**Figure 12:**
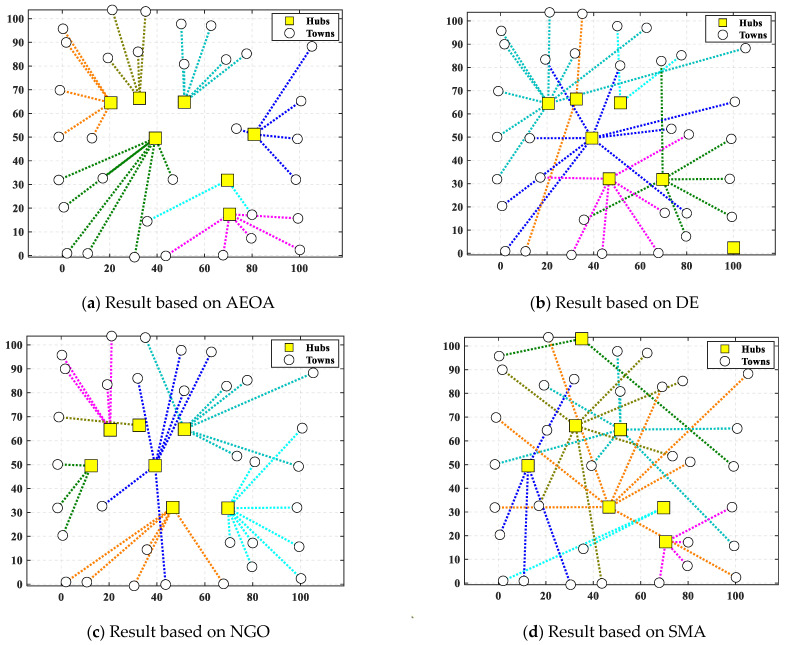
The diagram of best solutions based on different algorithms for case 2.

**Figure 13:**
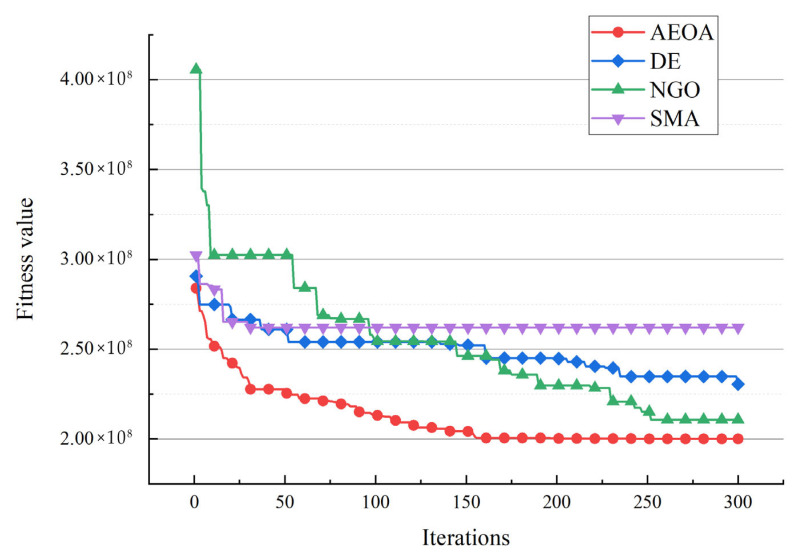
The iteration curves of different algorithms for case 2.

After the AEOA, the DE algorithm, the NGO algorithm, and the SMA had solved the problem, Table 10 and Figure 12 show the best solutions and the corresponding diagrams. Table 11 shows the fitness values of the best solutions obtained by various algorithms. For the trade hubs, based on the AEOA, towns 1, 2, 16, 20, 24, 28, and 32 are chosen. Combined with Figure 12, this scheme has a smaller construction cost than the other three algorithms. Meanwhile, the connection between towns and hubs of the solution based on the AEOA is more reasonable, having smaller transportation costs. For solutions based on the DE algorithm, the NGO algorithm, and the SMA, the connections between hubs and towns are more chaotic, leading to higher costs in transportation. The iteration curves in Figure 13 also illustrate that the AEOA has faster convergence speed and solving accuracy, meaning it is a suitable tool for such trade hub location and allocation problems.

(3) Trade hub location and allocation problem for main towns of Henan province

After being tested on two simulated numerical cases, the AEOA performs excellently in solving trade hub location and allocation problems among towns with different economic levels. Thus, the AEOA is employed to solve the trade hub location and allocation problem for main towns of Henan province mentioned in Section 2.1. Firstly, Table 1 lists the GDP of these main towns. As specified in Section 2, a higher economic level means that the cost of construction in this area is lower.

**Figure 14:**
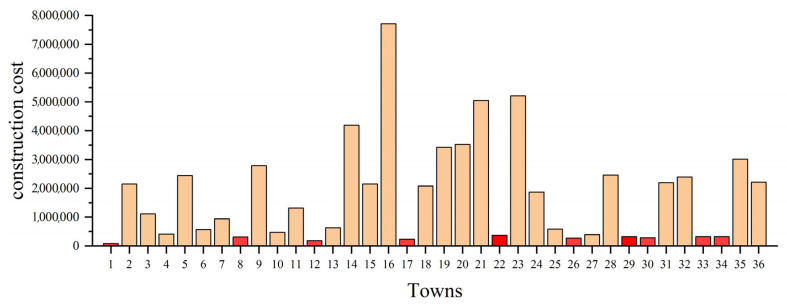
The construction costs of the main towns of Henan province.

Thus, Figure 14 shows the obtained construction cost of each town. It is calculated by multiplying the reciprocal of GDP by 1 × 10^9^. Those marked in red are the 10 towns with the lowest construction costs. In addition, the freight between towns is set to 5. The distance between towns is the mileage of the highway. Finally, based on the AEOA, the obtained best solution is shown in Table 12.

**Figure 15:**
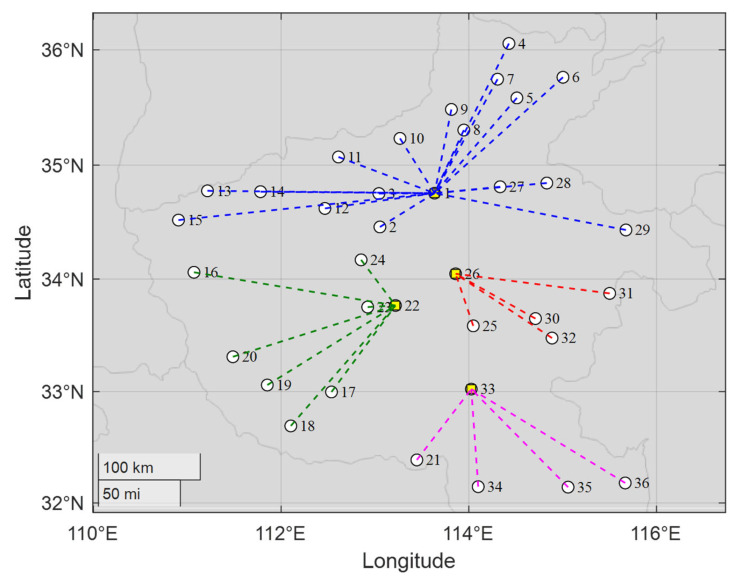
The diagram of best solutions based on the AEOA for the trade hub location and allocation problem for main towns of Henan province.

From Table 12, towns 1, 22, 26, and 33 are selected as suitable areas for construction, which belong to the towns with the lowest construction costs as shown in Figure 14. Meanwhile, since the freight between any two towns is the same, the remaining towns are connected to the nearest hubs to reduce the transportation costs, as shown in Figure 15. That is, the solution based on the AEOA is reasonable and suitable for achieving the aim of trade hub location and allocation. This method based on the AEOA is helpful and effective in assisting government departments in making decisions to accelerate local economic development.

## 6. Conclusions and Future Work

This paper proposes a trade hub location and allocation method based on a novel AEOA to facilitate the storage and low-cost transportation of goods between towns. Firstly, the trade hub location and allocation model is established. In the model, the construction and freight costs are regarded as the optimization targets, which are determined by a decision matrix with elements set to 0 or 1. When the choice of hubs is suitable, the lower freight between hubs and the reasonable connections between hubs and towns will effectively reduce the total cost. Then, a novel intelligent algorithm, AEOA, is proposed to solve the established model. In this algorithm, three main strategies achieve efficient search for complex decision spaces: the situational awareness and analysis stage, the free exploration stage, and the flight formation integration stage. According to the results on the CEC2022 test suite, the AEOA obtains solutions with higher accuracy for 11 functions out of all 12 functions. This illustrates that the proposed algorithm can search for potential solutions more effectively, approximating the theoretically optimal solution. This conclusion is also supported by the obtained *p* values of Wilcoxon rank sum test. Furthermore, based on the convergence and stability analysis, the AEOA can converge to better solutions within fewer iterations due to the combination of the free exploration stage and flight formation integration stage in artificial eagle populations. Finally, the AEOA is used to solve the established trade hub location and allocation model. Two simulated cases and a real case in Henan province, China, are solved to illustrate the characteristics of the proposed method. For the simulated cases, the AEOA can obtain smaller fitness values than other comparison algorithms, meaning its solution can reduce the cost of constructing hubs. The choice of hubs is more suitable because of the lower construction cost and reasonable connections between hubs and the rest of the towns. The results prove that the AEOA can handle various hub location and allocation problems. Thus, the proposed method selects suitable trade hubs among 36 key towns in Henan province. Government departments can use the results obtained for reference.

In the future, the AEOA may also be used for other complex engineering optimization problems, such as parameter optimization, resource scheduling, path planning, and image processing. The trade hub location and allocation method based on the AEOA can further be tested in actual cases in some economically developed areas.

## Figures and Tables

**Table 1:** The GDP of the main towns of Henan province in 2022 (in CNY 100 million).

No.	1	2	3	4	5	6	7	8	9
GDP	78,795.89	2,145,416.32	1,108,794.96	410,598.65	2,440,393.39	564,480.53	939,284.29	309,354.95	2,785,204.99
No.	10	11	12	13	14	15	16	17	18
GDP	467,981.43	1,311,947.73	183,583.15	631,897.30	4,189,183.53	2,147,166.81	7,714,263.67	230,297.02	2,079,477.64
No.	19	20	21	22	23	24	25	26	27
GDP	3,423,367.91	3,522,491.11	5,050,760.14	371,172.59	5,209,690.02	1,870,662.40	581,030.78	273,566.57	391,078.38
No.	28	29	30	31	32	33	34	35	36
GDP	2,458,452.16	324,325.48	286,022.20	2,193,078.64	2,392,058.37	324,378.48	326,268.13	3,014,318.01	2,208,285.49

**Table 2:** Information on the CEC2022 test suite.

Type	Functions	Description	Range	Dimension	*f*_min_
Uni-modal	F01	Shifted and Full Rotated Zakharov Function	[−100,100]	20	300
Multi-modal	F02	Shifted and Full Rotated Rosenbrock’s Function	[−100,100]	20	400
	F03	Shifted and Full Rotated Rastrigin’s Function	[−100,100]	20	600
	F04	Shifted and Full Rotated Non-Continuous Rastrigin’s Function	[−100,100]	20	800
	F05	Shifted and Full Rotated Levy Function	[−100,100]	20	900
Hybrid	F06	Hybrid Function 1 (*N* = 3)	[−100,100]	20	1800
	F07	Hybrid Function 2 (*N* = 6)	[−100,100]	20	2000
	F08	Hybrid Function 3 (*N* = 5)	[−100,100]	20	2200
Composition	F09	Composition Function 1 (*N* = 5)	[−100,100]	20	2300
	F10	Composition Function 2 (*N* = 4)	[−100,100]	20	2400
	F11	Composition Function 3 (*N* = 5)	[−100,100]	20	2600
	F12	Composition Function 4 (*N* = 6)	[−100,100]	20	2700

**Table 3:** Parameter settings of the comparison algorithms.

Algorithms	Parameters and Values
GJO	The constant *c*_1_ = 1.5.
HHO	The initial energy *E*_0_ = 2.
BWO	The probability of whale fall decreases from 0.1 to 0.05.
WO	Proportion of females *p* = 0.4.
CPO	Random step factor *DC* = 0.7.
PSO	Individual cognitive factor *c*_1_ = 2, social cognitive factor *c*_2_ = 2.5, acceleration weight *w* = 2.
NRBO	Deciding factor for trap avoidance operator *DF* = 0.6;
SCSO	The maximum sensitivity *S* = 2.

**Table 4:** Solving results of the AEOA and comparison algorithms on CEC2022.

F	Index	GJO	HHO	BWO	WO	CPO	PSO	NRBO	SCSO	AEOA
01	Ave_f	1.11 × 10^4^	3.61 × 10^2^	2.53 × 10^4^	9.26 × 10^2^	3.11 × 10^4^	4.16 × 10^3^	6.74 × 10^3^	8.13 × 10^3^	**3.00 × 10^2^**
	Std_f	3.00 × 10^3^	3.48 × 10^1^	6.03 × 10^3^	4.80 × 10^2^	8.13 × 10^3^	1.02 × 10^3^	1.99 × 10^3^	4.56 × 10^3^	**6.32 × 10^−4^**
	Rank	7	2	8	3	9	4	5	6	**1**
02	Ave_f	5.52 × 10^2^	4.66 × 10^2^	4.96 × 10^2^	4.55 × 10^2^	1.91 × 10^3^	4.87 × 10^2^	5.83 × 10^2^	5.04 × 10^2^	**4.24 × 10^2^**
	Std_f	5.32 × 10^1^	2.22 × 10^1^	1.80 × 10^1^	1.20 × 10^1^	3.81 × 10^2^	2.63 × 10^1^	5.80 × 10^1^	3.38 × 10^1^	**1.86 × 10^1^**
	Rank	7	3	5	2	9	4	8	6	**1**
03	Ave_f	6.15 × 10^2^	6.50 × 10^2^	6.11 × 10^2^	6.03 × 10^2^	6.83 × 10^2^	6.02 × 10^2^	6.46 × 10^2^	6.40 × 10^2^	**6.00 × 10^2^**
	Std_f	6.20 × 10	9.77 × 10	1.54 × 10	2.21 × 10	4.27 × 10	1.09 × 10	9.52 × 10	1.50 × 10^1^	**3.55 × 10^−2^**
	Rank	5	8	4	3	9	2	7	6	**1**
04	Ave_f	8.79 × 10^2^	8.78 × 10^2^	8.84 × 10^2^	8.77 × 10^2^	9.70 × 10^2^	8.99 × 10^2^	8.98 × 10^2^	8.84 × 10^2^	**8.72 × 10^2^**
	Std_f	2.24 × 10^1^	1.43 × 10^1^	1.04 × 10^1^	4.02 × 10^1^	8.99 × 10	1.50 × 10^1^	1.66 × 10^1^	1.97 × 10^1^	**2.01 × 10^1^**
	Rank	4	3	5	2	9	8	7	6	**1**
05	Ave_f	1.61 × 10^3^	2.63 × 10^3^	1.86 × 10^3^	1.36 × 10^3^	3.53 × 10^3^	1.02 × 10^3^	2.02 × 10^3^	2.21 × 10^3^	**9.00 × 10^2^**
	Std_f	3.87 × 10^2^	2.67 × 10^2^	5.22 × 10^2^	5.44 × 10^2^	2.99 × 10^2^	1.15 × 10^2^	3.80 × 10^2^	3.05 × 10^2^	**3.23 × 10^−1^**
	Rank	4	8	5	3	9	2	6	7	**1**
06	Ave_f	1.24 × 10^7^	6.52 × 10^4^	1.26 × 10^6^	6.15 × 10^3^	4.92 × 10^8^	1.55 × 10^5^	6.33 × 10^4^	7.77 × 10^5^	**3.72 × 10^3^**
	Std_f	1.94 × 10^7^	3.93 × 10^4^	7.28 × 10^5^	4.89 × 10^3^	2.46 × 10^8^	4.77 × 10^5^	2.25 × 10^5^	3.59 × 10^6^	**1.68 × 10^3^**
	Rank	8	4	7	2	9	5	3	6	**1**
07	Ave_f	2.08 × 10^3^	2.14 × 10^3^	2.07 × 10^3^	2.06 × 10^3^	2.22 × 10^3^	2.04 × 10^3^	2.14 × 10^3^	2.11 × 10^3^	**2.03 × 10^3^**
	Std_f	3.38 × 10^1^	4.61 × 10^1^	9.51 × 10	2.88 × 10^1^	2.66 × 10^1^	9.73 × 10	3.69 × 10^1^	3.08 × 10^1^	**7.75 × 10**
	Rank	5	8	4	3	9	2	7	6	**1**
08	Ave_f	2.24 × 10^3^	2.24 × 10^3^	2.23 × 10^3^	2.23 × 10^3^	2.48 × 10^3^	2.24 × 10^3^	2.29 × 10^3^	2.26 × 10^3^	**2.23 × 10^3^**
	Std_f	2.93 × 10^1^	2.22 × 10^1^	1.30 × 10	7.56 × 10	9.84 × 10^1^	2.27 × 10^1^	5.98 × 10^1^	3.87 × 10^1^	**9.67 × 10^−1^**
	Rank	4	6	3	2	9	5	8	7	**1**
09	Ave_f	2.54 × 10^3^	2.49 × 10^3^	2.49 × 10^3^	2.48 × 10^3^	2.91 × 10^3^	2.49 × 10^3^	2.55 × 10^3^	2.53 × 10^3^	**2.47 × 10^3^**
	Std_f	3.06 × 10^1^	4.12 × 10	3.53 × 10	2.56 × 10	9.06 × 10^1^	1.10 × 10	4.95 × 10^1^	3.62 × 10^1^	**9.86 × 10^−1^**
	Rank	7	3	5	2	9	4	8	6	**1**
10	Ave_f	3.45 × 10^3^	3.23 × 10^3^	2.52 × 10^3^	**2.52 × 10^3^**	3.31 × 10^3^	2.95 × 10^3^	3.79 × 10^3^	2.70 × 10^3^	2.52 × 10^3^
	Std_f	1.28 × 10^3^	6.81 × 10^2^	5.93 × 10^1^	**4.65 × 10^1^**	7.75 × 10^2^	4.57 × 10^2^	1.47 × 10^3^	5.36 × 10^2^	4.41 × 10^1^
	Rank	8	6	3	**1**	7	5	9	4	2
11	Ave_f	4.06 × 10^3^	3.00 × 10^3^	3.26 × 10^3^	3.01 × 10^3^	7.68 × 10^3^	3.01 × 10^3^	4.02 × 10^3^	3.35 × 10^3^	**2.91 × 10^3^**
	Std_f	4.45 × 10^2^	1.56 × 10^2^	1.11 × 10^2^	1.59 × 10^2^	5.30 × 10^2^	9.38 × 10^1^	4.29 × 10^2^	3.59 × 10^2^	**9.73 × 10^1^**
	Rank	8	2	5	4	9	3	7	6	**1**
12	Ave_f	2.99 × 10^3^	3.09 × 10^3^	2.96 × 10^3^	2.95 × 10^3^	3.13 × 10^3^	3.01 × 10^3^	3.01 × 10^3^	2.99 × 10^3^	**2.90 × 10^3^**
	Std_f	3.61 × 10^1^	1.19 × 10^2^	8.12 × 10	7.81 × 10	4.93 × 10^1^	1.56 × 10^1^	5.12 × 10^1^	3.33 × 10^1^	**1.57 × 10^−4^**
	Rank	4	8	3	2	9	6	7	5	**1**
Average rank		5.917	5.083	4.750	2.417	8.833	4.167	6.833	5.917	**1.083**
Final rank		6	5	4	2	9	3	8	6	**1**

**Table 5:** The results of Wilcoxon rank sum test between the AEOA and comparison algorithms.

CEC	GJO	HHO	BWO	WO	CPO	PSO	NRBO	SCSO
01	3.02 × 10^−11^/+	3.02 × 10^−11^/+	3.02 × 10^−11^/+	3.02 × 10^−11^/+	3.02 × 10^−11^/+	3.02 × 10^−11^/+	3.02 × 10^−11^/+	3.02 × 10^−11^/+
02	3.34 × 10^−11^/+	9.76 × 10^−10^/+	3.34 × 10^−11^/+	2.03 × 10^−9^/+	3.02 × 10^−11^/+	1.21 × 10^−10^/+	3.02 × 10^−11^/+	4.08 × 10^−11^/+
03	2.88 × 10^−11^/+	2.88 × 10^−11^/+	2.88 × 10^−11^/+	2.88 × 10^−11^/+	2.88 × 10^−11^/+	2.88 × 10^−11^/+	2.88 × 10^−11^/+	2.88 × 10^−11^/+
04	5.30 × 10^−1^/=	5.79 × 10^−1^/=	9.47 × 10^−3^/+	5.20 × 10^−1^/=	3.02 × 10^−11^/+	3.65 × 10^−8^/+	8.84 × 10^−7^/+	7.29 × 10^−3^/+
05	3.02 × 10^−11^/+	3.02 × 10^−11^/+	3.02 × 10^−11^/+	3.02 × 10^−11^/+	3.02 × 10^−11^/+	3.02 × 10^−11^/+	3.02 × 10^−11^/+	3.02 × 10^−11^/+
06	1.61 × 10^−10^/+	3.02 × 10^−11^/+	3.02 × 10^−11^/+	2.92 × 10^−2^/+	3.02 × 10^−11^/+	2.38 × 10^−7^/+	2.42 × 10^−2^/+	1.78 × 10^−4^/+
07	3.02 × 10^−11^/+	3.02 × 10^−11^/+	3.02 × 10^−11^/+	2.15 × 10^−6^/+	3.02 × 10^−11^/+	2.15 × 10^−6^/+	3.02 × 10^−11^/+	3.02 × 10^−11^/+
08	3.92 × 10^−2^/+	3.34 × 10^−11^/+	3.67 × 10^−3^/+	1.45 × 10^−1^/=	3.02 × 10^−11^/+	5.46 × 10^−9^/+	1.78 × 10^−10^/+	2.92 × 10^−9^/+
09	3.02 × 10^−11^/+	3.02 × 10^−11^/+	3.02 × 10^−11^/+	3.02 × 10^−11^/+	3.02 × 10^−11^/+	3.02 × 10^−11^/+	3.02 × 10^−11^/+	3.02 × 10^−11^/+
10	1.56 × 10^−8^/+	2.23 × 10^−9^/+	3.32 × 10^−6^/+	3.83 × 10^−6^/−	3.02 × 10^−11^/+	7.77 × 10^−9^/+	2.02 × 10^−8^/+	2.38 × 10^−7^/+
11	3.02 × 10^−11^/+	1.78 × 10^−4^/+	8.15 × 10^−11^/+	1.25 × 10^−4^/+	3.02 × 10^−11^/+	2.25 × 10^−4^/+	3.02 × 10^−11^/+	3.81 × 10^−7^/+
12	3.02 × 10^−11^/+	3.02 × 10^−11^/+	3.02 × 10^−11^/+	3.02 × 10^−11^/+	3.02 × 10^−11^/+	3.02 × 10^−11^/+	3.02 × 10^−11^/+	3.02 × 10^−11^/+
+/=/−	11/1/0	11/1/0	12/0/0	9/2/1	12/0/0	12/0/0	12/0/0	12/0/0

**Table 6:** The coordinates of each town in case 1.

No.	1	2	3	4	5	6	7	8	9	10	11	12	13	14	15	16	17	18	19	20
x	69.5	53.7	19.2	−0.3	48.4	52.7	2.0	71.8	98.9	79.4	69.9	99.9	1.4	29.0	44.6	2.6	95.7	107.5	53.5	3.5
y	98.1	69.8	5.3	18.1	28.2	48.5	44.6	54.6	99.8	17.7	1.7	51.5	5.3	21.9	94.5	77.1	29.0	73.6	5.3	100.9

**Table 7:** The best solution based on different algorithms for case 1.

	1	2	3	4	5	6	7	8	9	10	11	12	13	14	15	16	17	18	19	20
AEOA	8	6	5	5	—	—	5	—	8	5	5	8	5	5	6	6	8	8	5	6
DE	8	6	5	5	—	—	5	—	8	5	5	8	5	5	8	5	5	8	5	6
NGO	8	6	5	6	—	—	6	—	8	5	8	8	5	6	6	6	8	8	5	6
SMA	5	—	5	—	—	5	5	5	2	5	5	5	4	5	2	4	5	2	5	5

**Table 8:** The fitness values based on different algorithms for case 1.

	Transportation Cost	Construction Cost	Fitness Value
AEOA	9,402,315.2	8,114,591	55,126,167
DE	9,638,692.8	8,114,591	56,308,055
NGO	9,862,185.6	8,114,591	57,425,519
SMA	10,388,245	7,212,142	59,153,367

**Table 9:** The coordinates of each town in case 2.

No.	1	2	3	4	5	6	7	8	9	10	11	12	13	14	15	16	17	18	19	20
x	80.9	39.2	35.8	1.7	67.8	2.0	30.5	10.7	31.9	12.5	35.1	19.2	77.7	0.3	17.1	69.7	0.6	43.6	99.2	51.5
y	51.2	49.6	14.5	90.0	0.1	0.9	−0.7	0.9	86.0	49.5	103.1	83.4	85.2	95.7	32.6	31.8	20.4	−0.2	49.3	64.8
No.	21	22	23	24	25	26	27	28	29	30	31	32	33	34	35	36	37	38	39	40
x	99.4	73.4	−1.5	70.5	98.5	50.2	21.1	20.5	79.7	46.6	51.4	32.5	−1.5	105.3	100.7	80.0	−1.0	100.2	69.1	62.7
y	15.7	53.6	50.1	17.4	32.1	97.8	103.7	64.5	7.3	32.1	80.8	66.4	31.9	88.3	65.2	17.2	69.8	2.4	82.8	97.1

**Table 10:** The best solution based on different algorithms for case 2.

	1	2	3	4	5	6	7	8	9	10	11	12	13	14	15	16	17	18	19	20
AEOA	—	—	16	28	24	2	2	2	32	28	32	32	20	28	2	—	2	24	1	—
DE	30	—	16	28	30	2	30	32	28	2	32	2	20	28	30	—	2	30	16	—
NGO	16	—	30	28	30	30	30	30	2	—	20	28	20	28	2	—	10	2	20	—
SMA	30	20	16	32	24	16	10	10	10	—	—	20	32	11	32	—	10	32	11	—
	21	22	23	24	25	26	27	28	29	30	31	32	33	34	35	36	37	38	39	40
AEOA	24	1	28	—	1	20	32	—	24	2	20	—	2	1	1	16	28	24	20	20
DE	16	2	28	30	16	20	28	—	16	—	2	—	28	28	2	2	28	—	16	28
NGO	16	20	10	16	16	2	28	—	16	—	2	—	10	20	16	16	32	16	20	2
SMA	20	32	20	—	24	20	30	10	24	—	20	—	30	30	20	24	30	30	30	32

**Table 11:** The fitness values based on different algorithms for case 2.

	Transportation Cost	Construction Cost	Fitness Value
AEOA	34,451,386.4	27,773,090	200,030,000
DE	40,361,408.6	28,675,494	230,483,000
NGO	36,416,424.2	28,621,086	210,703,000
SMA	46,655,965.7	28,794,626	262,074,000

**Table 12:** The best solution based on the AEOA for the trade hub location and allocation problem for main towns of Henan province.

No.	1	2	3	4	5	6	7	8	9	10	11	12	13	14	15	16	17	18
AEOA	—	1	1	1	1	1	1	1	1	1	1	1	1	1	1	22	22	22
No.	19	20	21	22	23	24	25	26	27	28	29	30	31	32	33	34	35	36
AEOA	22	22	33	—	22	22	26	—	1	1	1	26	26	26	—	33	33	33

## Data Availability

All data generated or analyzed during this study were included in this published article.

## Appendix B. The Freight Between Different Towns in Case 1 and Case 2

**Table A1:** The freight between different towns in case 1.

No.	1	2	3	4	5	6	7	8	9	10	11	12	13	14	15	16	17	18	19	20
1	0	41	17	16	15	28	44	21	36	23	37	29	47	30	12	15	29	48	25	40
2	11	0	38	21	12	28	16	33	23	46	46	33	34	29	43	50	37	20	33	25
3	46	23	0	26	13	38	12	16	49	25	49	16	27	15	42	50	16	27	36	16
4	15	47	17	0	22	18	32	48	24	50	27	15	30	22	16	27	48	50	42	25
5	29	20	25	24	0	30	15	28	25	40	28	15	10	35	17	14	31	16	35	49
6	44	16	36	39	22	0	24	41	31	17	15	33	44	38	17	21	11	42	16	36
7	48	10	45	13	14	36	0	41	24	29	37	32	16	50	49	42	47	12	25	47
8	48	29	30	47	33	21	22	0	33	36	48	44	35	17	21	44	35	19	47	50
9	36	13	10	46	24	14	39	46	0	16	24	35	50	39	39	14	32	21	31	43
10	40	27	45	14	29	22	50	15	31	0	47	36	22	43	23	21	11	38	37	12
11	24	37	22	33	18	28	48	50	23	29	0	31	44	47	24	38	17	28	13	10
12	34	37	47	45	32	42	14	31	15	45	12	0	31	27	14	15	30	17	41	41
13	25	28	21	44	30	43	49	43	12	21	20	32	0	20	14	19	18	15	20	40
14	11	33	14	13	35	49	47	29	19	36	40	22	24	0	23	22	42	13	41	49
15	13	36	29	19	27	43	24	20	40	24	49	41	19	49	0	28	22	45	36	24
16	50	44	26	25	13	22	31	25	41	16	50	40	26	21	31	0	17	29	13	26
17	44	13	23	26	40	17	15	12	14	45	48	23	50	49	19	15	0	45	25	23
18	45	47	15	26	15	46	32	13	33	23	40	48	15	33	36	45	49	0	48	43
19	18	31	49	42	50	20	42	39	43	22	49	26	19	10	24	45	30	31	0	46
20	37	23	38	45	45	18	10	23	19	27	46	23	21	30	40	44	22	16	13	0

**Table A2:** The freight between different towns in case 2.

No.	1	2	3	4	5	6	7	8	9	10	11	12	13	14	15	16	17	18	19	20
1	0	42	36	21	17	50	39	13	50	45	37	17	30	35	38	46	34	33	13	14
2	16	0	33	49	18	20	35	49	38	44	14	10	23	34	31	24	16	42	21	11
3	37	32	0	21	44	30	17	45	43	28	25	47	47	12	16	44	30	40	34	12
4	31	19	11	0	48	41	36	40	45	29	13	45	50	30	12	23	49	15	13	26
5	31	28	23	15	0	27	21	21	36	34	46	33	21	28	30	16	39	44	28	45
6	24	23	19	24	14	0	46	30	35	48	20	19	49	47	12	30	33	35	47	34
7	26	21	36	28	38	32	0	31	50	13	50	50	15	34	24	30	30	24	35	11
8	11	12	46	50	36	20	25	0	50	45	49	22	36	40	17	42	33	30	35	21
9	34	12	49	28	16	33	46	26	0	24	14	38	37	23	28	16	35	26	16	44
10	13	25	15	20	13	43	34	27	42	0	16	24	19	21	13	36	26	37	46	29
11	26	49	16	17	33	43	40	20	18	33	0	10	34	30	39	32	20	23	42	19
12	24	34	46	14	49	33	39	28	17	15	50	0	25	42	32	45	43	12	13	48
13	47	30	32	25	28	21	18	37	12	24	28	24	0	27	24	35	33	22	10	15
14	40	34	12	37	37	27	30	21	35	46	50	35	10	0	20	31	31	44	48	16
15	10	41	32	16	10	13	50	30	48	20	25	38	26	45	0	42	39	44	14	37
16	37	12	11	15	22	38	35	44	50	34	15	12	49	39	41	0	49	15	11	32
17	14	19	45	42	28	42	15	41	24	19	12	41	42	13	12	14	0	41	36	46
18	20	13	46	40	24	10	23	44	50	17	46	25	47	19	31	41	25	0	26	28
19	27	40	18	17	39	44	24	14	14	16	20	35	14	22	40	40	48	26	0	30
20	13	31	39	23	17	18	47	14	34	13	45	13	14	14	48	31	17	21	39	0
No.	21	22	23	24	25	26	27	28	29	30	31	32	33	34	35	36	37	38	39	40
1	14	50	47	22	27	26	48	45	38	37	22	20	47	16	40	19	22	36	20	22
2	27	20	39	15	32	43	47	20	19	49	16	21	14	39	10	34	27	27	39	47
3	38	43	18	47	10	24	33	49	17	37	33	39	28	28	18	10	16	44	17	37
4	17	47	45	10	44	50	48	20	43	40	28	44	38	34	37	15	24	27	28	41
5	44	36	19	28	18	32	27	45	45	14	36	44	37	50	21	12	31	12	34	16
6	38	45	38	32	41	17	19	32	38	16	30	26	13	12	26	19	14	48	14	44
7	35	10	26	24	27	46	41	18	39	47	26	32	39	25	45	13	29	24	23	45
8	17	30	26	36	35	50	48	10	21	20	33	12	50	38	15	27	40	41	37	46
9	47	47	33	39	41	41	11	18	47	34	13	39	48	37	15	32	50	10	11	15
10	35	25	31	48	23	43	29	13	50	14	12	25	18	44	49	26	38	14	48	35
11	20	17	39	34	11	12	24	15	29	29	11	32	18	19	35	29	38	16	18	29
12	49	43	33	42	12	36	43	44	18	43	49	31	38	36	29	28	30	12	26	49
13	35	17	26	34	27	12	30	25	22	27	23	42	23	44	25	47	10	32	18	26
14	24	47	46	50	31	17	48	47	16	44	45	22	18	19	50	23	24	45	13	16
15	18	49	25	26	41	45	38	16	35	22	49	27	32	10	45	41	46	33	12	50
16	42	11	29	43	33	43	14	47	31	26	10	17	11	25	45	14	23	27	29	31
17	20	45	46	42	15	19	21	38	43	31	23	36	39	11	41	21	18	39	19	13
18	18	15	24	46	41	23	17	18	27	24	48	45	26	45	17	24	41	25	39	29
19	48	46	40	26	47	27	28	10	33	18	28	20	29	36	23	45	10	36	14	31
20	30	48	48	31	10	48	23	26	23	11	32	19	38	28	17	27	47	39	32	25
No.	1	2	3	4	5	6	7	8	9	10	11	12	13	14	15	16	17	18	19	20
21	14	50	47	22	27	26	48	45	38	37	22	20	47	16	40	19	22	36	20	22
22	27	20	39	15	32	43	47	20	19	49	16	21	14	39	10	34	27	27	39	47
23	38	43	18	47	10	24	33	49	17	37	33	39	28	28	18	10	16	44	17	37
24	17	47	45	10	44	50	48	20	43	40	28	44	38	34	37	15	24	27	28	41
25	44	36	19	28	18	32	27	45	45	14	36	44	37	50	21	12	31	12	34	16
26	38	45	38	32	41	17	19	32	38	16	30	26	13	12	26	19	14	48	14	44
27	35	10	26	24	27	46	41	18	39	47	26	32	39	25	45	13	29	24	23	45
28	17	30	26	36	35	50	48	10	21	20	33	12	50	38	15	27	40	41	37	46
29	47	47	33	39	41	41	11	18	47	34	13	39	48	37	15	32	50	10	11	15
30	35	25	31	48	23	43	29	13	50	14	12	25	18	44	49	26	38	14	48	35
31	20	17	39	34	11	12	24	15	29	29	11	32	18	19	35	29	38	16	18	29
32	49	43	33	42	12	36	43	44	18	43	49	31	38	36	29	28	30	12	26	49
33	35	17	26	34	27	12	30	25	22	27	23	42	23	44	25	47	10	32	18	26
34	24	47	46	50	31	17	48	47	16	44	45	22	18	19	50	23	24	45	13	16
35	18	49	25	26	41	45	38	16	35	22	49	27	32	10	45	41	46	33	12	50
36	42	11	29	43	33	43	14	47	31	26	10	17	11	25	45	14	23	27	29	31
37	20	45	46	42	15	19	21	38	43	31	23	36	39	11	41	21	18	39	19	13
38	18	15	24	46	41	23	17	18	27	24	48	45	26	45	17	24	41	25	39	29
39	48	46	40	26	47	27	28	10	33	18	28	20	29	36	23	45	10	36	14	31
40	30	48	48	31	10	48	23	26	23	11	32	19	38	28	17	27	47	39	32	25
No.	21	22	23	24	25	26	27	28	29	30	31	32	33	34	35	36	37	38	39	40
21	0	27	47	42	18	23	39	22	41	24	25	25	17	50	11	10	32	47	46	36
22	25	0	36	40	15	20	45	48	12	15	47	19	17	14	50	12	22	36	19	23
23	18	27	0	20	31	24	11	24	46	45	42	33	21	10	27	35	24	47	10	30
24	43	40	29	0	26	41	26	41	47	15	21	38	28	31	50	13	26	15	31	49
25	29	49	42	23	0	26	47	30	40	21	14	43	40	48	31	47	49	39	34	12
26	17	22	13	26	15	0	41	24	49	22	35	42	29	37	47	22	15	38	21	36
27	45	32	50	43	36	12	0	13	41	42	28	18	16	39	41	37	45	45	48	43
28	48	49	16	39	33	26	19	0	27	21	30	41	10	50	28	10	11	36	22	43
29	29	35	11	16	37	50	22	48	0	17	41	35	10	20	31	11	11	41	26	12
30	49	14	31	17	50	33	14	30	34	0	25	49	48	22	17	30	25	26	20	45
31	28	16	50	32	48	38	40	43	22	28	0	34	10	15	33	18	27	47	40	15
32	32	38	44	19	28	13	17	42	15	36	31	0	20	33	41	46	40	21	32	26
33	28	47	39	24	45	12	38	24	36	24	22	29	0	31	35	23	25	10	36	20
34	35	16	39	15	27	27	34	31	38	25	49	50	41	0	19	29	19	27	29	22
35	16	47	20	48	32	47	41	16	40	17	18	23	31	48	0	45	14	13	13	23
36	39	18	40	18	32	11	41	48	10	47	48	16	48	42	17	0	13	10	38	10
37	40	24	22	17	23	30	30	21	35	24	28	40	40	12	28	10	0	24	49	29
38	22	37	26	37	36	33	33	39	24	10	13	49	43	37	43	43	42	0	36	44
39	29	48	15	27	37	40	50	44	27	16	27	27	43	32	26	31	44	29	0	15
40	11	17	31	23	43	17	23	45	31	28	39	24	22	12	47	23	21	34	46	0
